# Consistency and validity of the inventory of callous- unemotional traits in a multi-centric community sample

**DOI:** 10.1016/j.heliyon.2022.e09789

**Published:** 2022-06-26

**Authors:** Olber Eduardo Arango-Tobon, Gabriel David Pinilla-Monsalve, Andrés Mauricio Grisales-Aguirre, Anyerson Stiths Gómez-Tabare, César Andrés Carmona-Cardona

**Affiliations:** a Luis Amigó Catholic University, Faculty of Psychology, Basic and Applied Neuroscience Research Center, Transversal 51A No. 67B 90, 050035, Medellín, Colombia; b Icesi University, Faculty of Law and Social Sciences, Department of Psychological Studies, Calle 18 No. 122-135, 760031, Cali, Colombia; c Luis Amigó Catholic University, Faculty of Management, Economics and Accounting Science, Basic Sciences Research Center, Transversal 51A No. 67B 90, 050035, Medellín, Colombia

**Keywords:** Adolescent, Callous-unemotional, Child, Psychopathy, Validity

## Abstract

The purpose of this research was to validate the Inventory of Callous Unemotional Traits in a multi-centric community sample of Colombian children and adolescents aged between 9 and 18 years. An adapted version to the Colombian Spanish was applied to 903 school students without significant medical background (neurotypical behavior), and 118 with a clinical history of internalizing or externalizing conditions. A group of specialized judges approved the content validity of the instrument in terms of relevance and intelligibility, but concept factorial validity was low for the uncaring and callousness factors. Exploratory factor analysis confirmed the existence of three dimensions (uncaring, unemotional, and callousness), but only 17 out of 24 items demonstrated adequate psychometric statistics. The consistency for the 17-item Colombian adaptation was acceptable (α = .78). Goodness-of-fit calculated through confirmatory analysis was satisfactory for a bifactor structure (model C). Neurotypical participants showed lower total scores in comparison to the other groups. Participants with internalizing conditions had higher unemotional traits, while those with externalizing behaviors more commonly presented uncaring behaviors. This study is important for psychopathy research in Colombia as provides a validated adaption of the most used instrument to assess callous-unemotional traits in children and adolescents.

## 1 Introduction

Psychopathy has been understood as a multidimensional construct involving behavioral, interpersonal, and emotional characteristics that configure a personality pattern and affect individual social functioning (Hare et al., 1991). Traditionally, psychopathy has been assessed in samples of adults with a history of criminal conduct and a special emphasis on the interpersonal/affective features related to personality (Cleckley, 1941, 1976). Nevertheless, this focus was reformulated with the development of the Psychopathy Checklist in its reviewed version (PCL-R, Hare et al., 1991), by the description of two interrelated factors. The first one refers to an interpersonal and affective profile associated with psychopathic personality features (lack of guilt and remorse, affective insensibility, lies, and instrumentalization) while the second one, describes a particular lifestyle (social instability, parasitism, seek of sensations, poor behavioral control, and impulsivity) (Hare, 1985).

The bi-dimensional structure of PCL-R has shown strong associations with violence, antisocial behaviors, and juvenile delinquency, which raised an interest regarding the existence of childhood and adolescence factors that could predict psychopathy in adults (Kotler and McMahon, 2010). Longitudinally, psychopathic features originated during childhood can be the expression of different psychiatric illnesses characterized by disruptive or externalizing behaviors including attention-deficit/hyperactivity disorder, oppositional defiant disorder, and conduct disorder (Frick et al., 2014).

In the 1990s, awareness of psychopathic traits in children and adolescents significantly increased and some studies suggested that, beyond its existence, these traits could configure a stable pattern of personality in adults (Harpur and Hare, 1994; Lynam, 1996). Unfortunately, this concept lacked adequate empirical evidence as the instruments implemented to identify psychopathic features in minors had been created to evaluate delinquent adults (Edens et al., 2001). As a consequence, some researchers centered their studies on the development of specific measures to evaluate psychopathy in children and adolescents, initially considering two perspectives. The first was supported by the correlation between conduct disturbances and severe antisocial behavior in children and led to the design of the Child Psychopathy Scale, which is a 41-item instrument that evaluates behavioral impairments and hyperactivity-impulsivity with attention deficit (Lynam, 1996). The second line of research proposed that additionally to the existence of abnormal conduct, psychopathy in children could be understood from the interaction of narcissism and impulsivity with deficits in emotional expression and experiencing (callous-unemotional, CU) (Barry et al., 2000). Combining these scopes, the Antisocial Process Screening Device (APSD, Frick and Hare, 2001) was designed to assess psychopathic traits from three dimensions (narcissism, impulsivity, and CU), and comprises 20 items equivalent to those in the PCL-R, using reports from the child, his/her parents, and teachers (Frick et al., 1994). The APSD has been used as a research instrument to analyze the presence and development of psychopathic traits and antisocial behaviors in children and adolescents (Pechorro et al., 2014a). However, concerns about the reliability of self-reporting (particularly on the CU subscale) motivated the elimination of items with low statistical performance to improve validity and internal consistency.

In order to overcome the validity flaws observed with the APSD and its CU subscale, Frick et al. (2004) developed the Inventory of Callous-Unemotional Traits (ICU). Originally, this scale was constructed to evaluate the CU dimension in children and adolescents as a unidimensional psychopathy factor relative to callousness, based on the four items included in the APSD for that purpose (“is concerned about how well she/he does in school or work”, “feels bad or guilty when she/he does something wrong”, “is concerned about the feelings of others”, “does not show feelings or emotions”). Six items were developed from each of the previous statements, half of them written in a positive direction, for a total of 24 items. Responses are codified on a 4-level Likert format from 0 (“not at all true”) to 3 (“definitely true”). ICU considers three reporting versions: parents, teachers, and self-report (Cardinale and Marsh, 2020). Essau et al. (2006) conducted the first validation study of the ICU by analyzing the self-report version in a community sample of 1443 German adolescents aged 13–18 years. Results of the confirmatory factor analysis indicated a bifactor structure with adequate goodness of fit (df = 200, χ2 = 935.53, GFI = .90, AGFI = .85, RMSEA = .07), depicting a general factor of psychopathic traits (total score) and a specific factor that nested three subscales relative to reduced empathic responses (callousness, 11 items), lack of concern for performance and relationships with others (uncaring, 8 items), and poor emotional expressions and experiencing (unemotional, 5 items). In general, the ICU showed an acceptable internal consistency for its total score (Cronbach's α = .77), and the callousness (α = .70) and uncaring (α = .73) subscales. The unemotional dimension showed the worst estimate (α = .64) and the lowest correlations with the total score and the other subscales (callousness Pearson's ρ = .25; uncaring ρ = .09).

The promising results by Essau et al. (2006) showed that the psychometric properties of the ICU support its efficiency and validity for measuring psychopathic traits in children and adolescents; even though, it is necessary to mention some structural elements that limit its reliability. The internal consistency and correlations of the unemotional scale with the total score and the other subscales present low and marginal estimates, which might imply that the unemotional factor is not significantly contributing to the CU construct and could possibly be an independent dimension that should be evaluated separately; furthermore, the few items included in this subscale (5 statements) and their direction (3 negatives and 2 positives) could bias the correlation statistics and its consistency. Likewise, it is important to account for the linguistic adaptation processes to non-English languages and the version of the instrument (parents, teachers, or self-report), as these may partially influence the consistency and validity of the scale.

Subsequent factorial validations of the ICU using reports from children, parents, and teachers in clinical or mixed samples and with diverse cultural or demographic characteristics corroborated the bifactor model found by Essau et al. (2006) but also invariably pointed out the substandard psychometric properties of the unemotional subscale (Ciucci et al., 2014; Ezpeleta et al., 2013; Kimonis et al., 2008, 2014; Fanti et al., 2009; Roose et al., 2010).

Few validations have been performed in Hispanic populations. In the Spanish community sample (n = 138) analyzed by López-Romero et al. (2015a), internal consistency was better for the total score (α = .76) than for the callousness dimension (α = .58). In this study, estimates were the lowest for the unemotional factor (α = .50), but this was the dimension with the highest internal consistency. All the correlations among subscales were significant. The same research group (López-Romero et al., 2015b) examined the factorial structure in institutionalized youths (n = 324) finding that loadings for items 2, 10, and 12 were lower than the threshold defined. They selected a hierarchical model as the best after removing item 10 (SRMR = .07, AGFI = .95) and disregarded a possible influence of the items’ wording direction. Moreover, Romero & Alonso (2017) determined a high total and subtotal Cronbach α, and significant correlations between the subscales and personality traits according to the Five Factors Model: callousness-agreeableness, uncaring-conscientiousness, and unemotional-extraversion.

The closest population to the South-American context, is the Mexican sample (n = 679) studied by Galvan-García (2011), who found a total score consistency of .76 (uncaring α = .69, unemotional α = .59, callousness α = .66). The estimate increased in a subsample of male participants in conflict with the law (α = .77), especially for the callousness subscale (α = .66). These Cronbach's α are lower than those found in non-Latin American samples, which might imply that the callousness subscale reliability partially varies according to culture. Related to the consistency of the items, Galvan found low factorial loadings (<.40) for items 2 and 5; they also obtained higher loadings in item 8 for the uncaring subscale and in item 10 for the unemotional subscale, while both theoretically belong to the callousness factor.

Considering the results from this factorial analysis in Mexican children and adolescents, Amador et al. (2017) removed multiple questions obtaining a shorter version with 13 items but higher internal consistency (total score α = .74). This version comprises 6 items for callousness (original items 7, 9, 11, 12, and 21; α = .74), 4 for uncaring (items 15, 16, 17, and 23; α = .71) and 3 for unemotional (1, 14, and 19; α = .66). In a larger sample, Amador-Zavala and Padrós-Blázquez (2019) validated the 13-item version (α = .67) in 758 youths (12–22 years), calculating a three-factor model with satisfactory goodness of fit statistics (RMSEA = 0.04, SRMR = 0.04). Their study demonstrates a low-moderate correlation degree between the three subscales and the absence of significant covariance between callousness and unemotionality.

A recent meta-analysis conducted by Deng et al. (2019), grouped 146 studies with 64,356 individuals finding a mean Cronbach's α of .81 (95% CI .80 - .82) for the total score, .70 (95% CI .57 - .73) for unemotional, .78 (95% CI .77 - .80) for uncaring, and .75 (95% CI .73 - .77) for callousness. Across moderators, higher consistencies were calculated for combined self-report/parent-report version, infant and young children, and offenders. These results are coherent with that obtained in the meta-analytic review by Cardinale and Marsh (2020) who analyzed 75 studies and 115 samples for a total of 27,947 individuals. Their findings support an acceptable internal consistency (total score α = .83, uncaring α = .80, callousness α = .75, unemotional α = .79) with a correlation effect size that was moderate-large for callousness-uncaring (ρ = .45) and small-moderate for uncaring-unemotional (ρ = .39) and callousness-unemotional (ρ = .24). Concurrent validity was significant with measures of the behavioral, interpersonal, and affective facets of psychopathy.

Timely diagnosis of CU during childhood is needed for early interventions that prevent violence committed by individuals with disruptive conduct (Kotler and McMahon, 2005), and hence more evidence is required to confirm the validity of the proposed CU factors across different cultures, languages, and populations. Colombia is a South-American country that struggled with a 50-years-long internal conflict and is currently facing high rates of armed violence that is not alien to children and adolescents. Minors recruited by guerrillas have participated in shootings (40%), killings (18%), and kidnaps (13%) (Bácares-Jara, 2015); in the cities, young civilians are not infrequently charged with domestic violence, theft, personal injuries, and property damage (Morales-Ortega and Castillo-Bolaño, 2011). Even so, no psychological instrument has been strictly validated in the country to analyze the levels of CU in children and adolescents, regardless of their offender status.

The objective of this research is to examine the internal consistency and validity of the ICU in Colombia. We expected to identify a good consistency for the total score and to recognize the same three factors (uncaring, unemotional, and callousness) suggested by Essau et al. (2006). Additionally, it was hypothesized that the best structure corresponds to a bifactor model and that the total ICU score accurately discriminate students with a medical history of externalizing behaviors from those who appear to be neurotypical in the community settings.

## 2 Method

This is a psychometric study with the purpose of validating a translated version of the ICU in a multi-centric sample of Colombian children and adolescents. Content validity was confirmed by a group of judges who rated the relevance and intelligibility of the items. Multidimensional reduction was accomplished through the principal-axis method and confirmatory factor analysis. Besides, criterion validity for distinguishing participants with externalizing behaviors was established with a logistic model. The recruitment and identification of students with internalizing or externalizing disorders were carried out through the use of a Google Forms questionnaire, in which parents were asked if their children had any psychological, psychiatric, or neurological diagnosis.

### 2.1 Sample size and selection

For validating the ICU, we calculated a minimum community sample of 240 participants considering 10 individuals for each of the items proposed by the ICU (Boateng et al., 2018). Additionally, to pursue a confirmatory factor analysis, it has been suggested a minimum of 20 observations for each observed and latent variable (24 items, 3 dimensions, 1 general construct), and therefore the final sample size was set to ≥560 participants (Kline, 2015).

Randomized sampling was not applied as we included every individual fulfilling the main inclusion criterion (4^th^-11^th^ grade children and adolescents, who are usually aged 10–18 years in Colombia). For construct validation, those with psychiatric and neurological conditions diagnosed by a licensed physician or psychologist and reported by their parents or teachers were excluded.

### 2.2 Participants

We included 903 neurotypical children and adolescents, for evaluating reliability and construct validity. Moreover, 54 participants with a referred clinical history of internalizing conditions (depression, anxiety, and/or bipolar disorder) and 64 students with externalizing pathologies (attention-deficit/hyperactivity, oppositional defiant, and/or conduct disorder) (Cardinale and Marsh, 2020) were studied to determine the criterion validity (Table 1).

**Table 1:** Main sociodemographic characteristics of the studied participants.

Group	Male sex	Age (IQR)	Grade (IQR)	Private school
Neurotypical (n = 903)	541 (59.91%)	14 (12–16)	8 (6–10)	761 (84.27%)
Internalizing (n = 54)	17 (31.48%)	14 (14–16)	9 (8–10)	49 (90.74%)
Externalizing (n = 64)	53 (82.81%)	14 (12–16)	8 (7–9)	59 (92.18%)

The included participants pertain to different socioeconomic levels and studied in six schools located in six different Colombian municipalities (Madrid, Cundinamarca; Bucaramanga, Santander; Manizales, Caldas; Palmira, Valle del Cauca; Bello and La Estrella, Antioquia). School selection was random and influenced by the current COVID-19 pandemics as we could only include institutions that had had previously agreed to collaborate with our group for past research. Ethics approval was provided by Luis Amigó Catholic University Institutional IRB (No. 62888/2021).

### 2.3 Procedure

Idiomatic adaptation of the ICU to Colombian Spanish fulfilled the following stages: translation from the original version by Frick (2004) by two Colombian natives, bilingual in Spanish and English; reconciliation of both versions by the research team; reverse translation by two English native speakers with bilingual proficiency in Spanish; second reconciliation by the research team; and comparison of the obtained version with the original instrument. Similarity (exact coincidence, minor changes, and related meaning) was assessed using CopyLeaks Checker (Yamin and Bitton, 2013).

Content validity regarding the relevance and intelligibility of the adapted version was analyzed by 20 Colombian experts in the field (i.e., researchers, university professors, and/or clinicians) (Rubio et al., 2003), who scored both properties in a 5-options Likert scale. A JotForm 4.0. file (Tank, 2017) was used for recording the answers and presented the judges with the purpose of the study, briefly provided the current research context of CU, and incorporated the basic definitions of uncaring, unemotional, and callousness dimensions stated by Essau et al. (2006). in their original paper.

After receiving the approval of the schools, parents and teachers were told this study aimed to validate an instrument that characterizes children's emotions in different situations in their daily life. Parents provided written consent for participating in the study and students were also asked for their assent. Collaborating teachers were explained the purpose of the investigation and provided with specific details about the application procedure for guaranteeing assistance and independent responses. ICU was asynchronously applied, and responses were submitted directly by the students in a Google Forms file (Google, 2017). Data collection was completed in two months and the procedure was the same for all students regardless of their conduct type (neurotypical, externalizing, or internalizing).

### 2.4 Statistical analysis

The total score was calculated after reverse scoring of items 1, 3, 5, 8, 13, 14, 15, 16, 17, 19, 23, and 24. Central tendency and dispersion measures were informed according to the data distribution evaluated using Shapiro-Wilk and D'Agostino tests. Categorical data were reported with absolute and relative frequency. Atypical ICU total scores were studied using the ROUT method and assigning a 1% Q coefficient. For defining the presence of bivariate significant statistical differences, Mann-Whitney's U and Kruskal-Wallis tests with Dunn's posthoc and Sidak correction were applied. Correlations were based on Spearman's ρ coefficient.

Content validity was investigated using an index derived from the coefficient validity ratio (CVR) (Lawshe, 1975), with and without correction for the number of judges (experts) (Tristan, 2008). As CVR requires three possible responses (essential, useful but expendable, and unnecessary), we grouped Likert options 4 and 5 as essential, and 1 and 2 as unnecessary. For reducing the possible bias introduced by a judge, we calculated the coefficient of content validity (CVC) proposed by Herná ndez-Nieto, 2002. As ICU seems to be a multidimensional psychometric instrument, the index of factorial validity (IFV) was studied for each item and by construct (Rubio et al., 2003).

Reliability was studied with Cronbach's α coefficient. To determine ICU construct validity, exploratory factor analysis was performed with the principal-axis method for factoring extraction after identifying the suitability of the data with the Kaiser-Meyer-Olkin measure and Bartlett's test of sphericity. Factors were extracted if located to the left of the first significant elbow in the Cattell scree plot (Zwick and Velicer, 1982); factor loadings were then rotated with the *oblimin* method without Kaiser normalization. Items were retained if these features were observed: a) the highest loading was found for the same dimension suggested by the original research, b) maximum factor loading ≥.40, and c) differences between factor loadings for each dimension ≥≈ .20. Spearman correlation matrix with Benjamini and Hochberg correction for false discovery was calculated to examine convergent and divergent validities.

The factorial structure was confirmed by considering a theoretical covariance between the three-dimension for a maximum of 4 latent and 24 observed variables. As ICU provides ordinal responses, parameters estimation was performed with the weighted least-squares method (asymptotic distribution-free) or with the maximum likelihood strategy when no convergence was achieved with the first. The goodness of fit statistics (i.e., Akaike (AIC) and Bayesian information criteria (BIC), root mean square error of approximation (RMSEA), standardized root mean square residual (SRMR), comparative fit index (CFI), Tucker-Lewis index (TLI), etc.) were used for the comparison of possible models. The invariance of the model by sex was studied as well.

To verify the criterion validity, a multinomial logistic regression model was built for determining which items and dimensions most contribute to discriminating between neurotypical, externalizing, and internalizing conduct. The best total score cut-off to differentiate participants with externalizing disorders from neurotypical students was calculated with the Youden's index and relevant diagnostic statistics were reported. Significance was established if p < .050.

According to Herrera (1998), the total validity of an instrument can be measured as the average of content, construct, and criterion validity. Some researchers have proposed that Cronbach's α is a statistic for criterion validity but according to other authors is a way to support the structure of an instrument (Tavakol and Dennick, 2011). For the purpose of this study, total validity was computed as the average of Lawshe's coefficient validity index with Tristan's adjustment (content validity), adjusted Cronbach's α (internal consistency), goodness of fit index (construct validity), and area under the ROC curve (criterion validity). Analyses were made on R 4.1.0 (Ihaka and Gentleman, 2021) and Stata 16 (Gould, 2019).

## 3 Results

### 3.1 Idiomatic adaption of the inventory

The Colombian Spanish version has 204 words with a similarity of 83.40% when its translation is compared to the original instrument by Frick et al. Identical words represented the 72.70% (149). There were 3.40% of minor changes and 7.30% of words with related meanings. Items 15 and 16 from the uncaring factor and item 21 from the callousness dimension showed the highest discrepancies. The final adapted version can be found in the Supplementary File.

### 3.2 Content validity

Every judge supported the apparent validity of the test. The median Likert score for relevance was 4.8 (IQR 4.5–4.9) and the CVR for the complete instrument was .81 (adjusted to .91 using Tristan's formula); the lowest ratios were found for items 7 and 20 (.40, adjusted .70). CVC was 1 (IQR .90 - 1). Nonetheless, IFV only reached .47 for uncaring, .93 for unemotional, and .40 for callousness. Intelligibility was scored 4.8 (IQR 4.6–4.9).

### 3.3 Acceptability and description of scores in neurotypical participants

**Figure 1:**
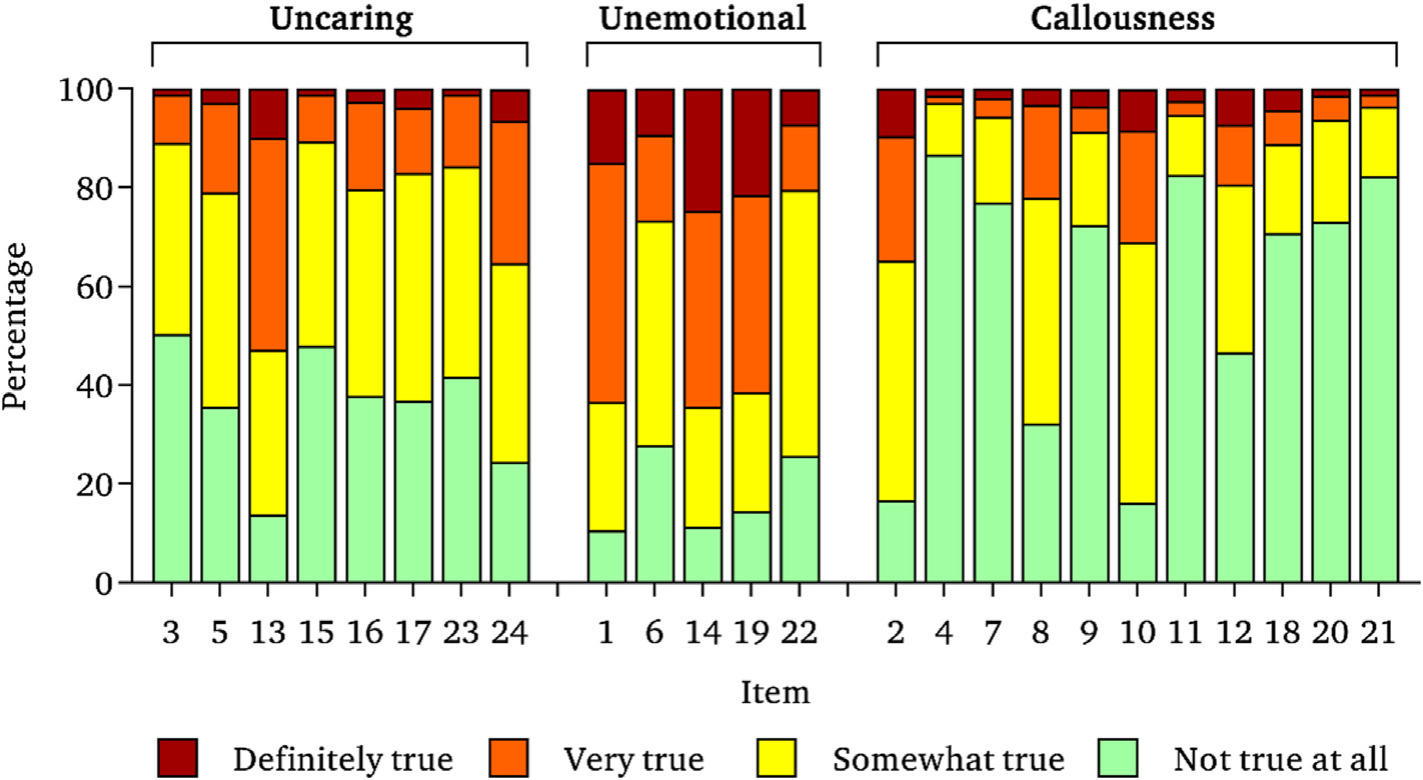
Relative frequency of Likert responses by item and factor.

Full computability of the data was achieved and none of the total scores was found as an outlier. Median total score was 20 (IQR 15–26), and showed a non-normal (W = 0.99, p < .001), asymmetrical (χ^2^ = 17.85, p < .001), but approximately mesokurtic (χ^2^ = 17.85, p = .729) distribution. The difference between the median and mean was 1.73% of the maximum achieved score (49). The floor effect was 43.16% (IQR 10.74–86.60) and the ceiling effect was not relevant as the response representing the highest level of CU was selected by the 6.28% (IQR 1.11–24.81). The distribution of Likert responses by item is presented in Figure 1.

Similar scores by sex were observed (W = 102803, p = .203) but there was a positive and significant correlation with age (Spearman's ρ = .29, p < .001) and grade (ρ = .26, p < .001). No correlation was observed with socioeconomic status (ρ = .02, p = .529) and, coherently, there were no differences between students from public or private schools (W = 49815, p = .139).

### 3.4 Reliability and construct validity

The original instrument exhibited a Cronbach's α of .81 (inter-item covariance of .09). Lower item-rest correlations were found for items 2 and 10. After their elimination, reliability only increased by 0.60% and 1.35% (Table 2). Cronbach's α was .76 for uncaring, .77 for unemotional, and .58 for callousness.

**Table 2:** Internal consistency of ICU items.

Item	Item-test correlation	Item-rest correlation	Inter-item covariance	Cronbach's α
1. Affective expression	0.573	0.495	0.087	0.792
2. Discernment	0.105	-0.001	0.100	0.818
3. Concern for school duties	0.488	0.416	0.091	0.797
4. Utilitarianism	0.366	0.310	0.095	0.803
5. Guilt	0.434	0.349	0.091	0.800
6. Emotional display	0.484	0.391	0.089	0.798
7. Punctuality	0.336	0.265	0.095	0.804
8. Affective interest	0.531	0.455	0.089	0.795
9. Potential for conflict	0.273	0.184	0.096	0.808
10. Emotional self-control	0.210	0.111	0.097	0.812
11. Academic performance	0.301	0.228	0.095	0.805
12. Coldness and disinterest	0.509	0.418	0.088	0.797
13. Fallibility	0.399	0.305	0.092	0.803
14. Affective identification	0.479	0.381	0.089	0.799
15. Perseverance	0.525	0.458	0.090	0.796
16. Pride	0.588	0.518	0.087	0.792
17. Affective intention	0.535	0.459	0.089	0.795
18. Remorse	0.323	0.230	0.094	0.806
19. Emotionality	0.584	0.497	0.085	0.792
20. Dedication	0.385	0.315	0.094	0.802
21. Affective irrelevance	0.426	0.369	0.094	0.800
22. Affective dissimulation	0.551	0.474	0.088	0.794
23. Tenacity	0.492	0.418	0.090	0.797
24. Kindness	0.403	0.307	0.092	0.803

**Figure 2:**
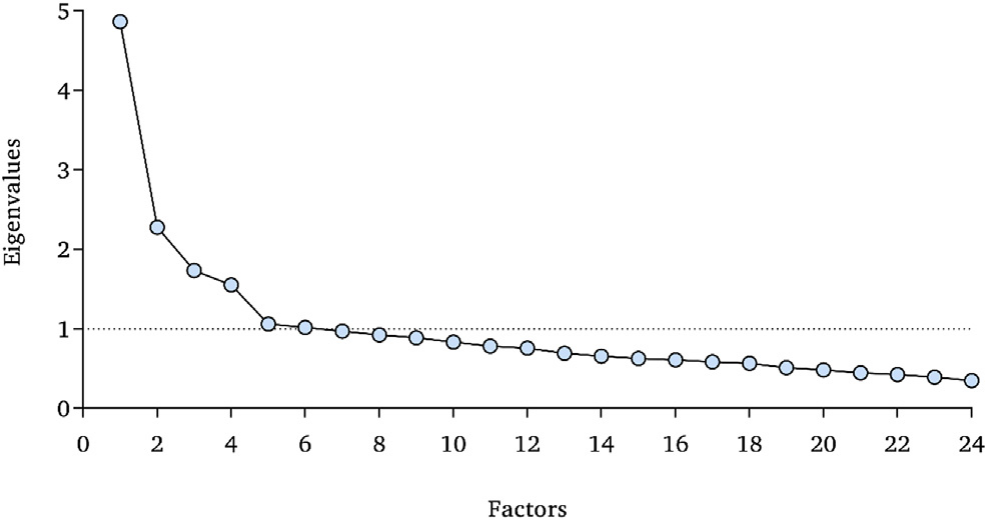
Eigenvalues by number of factors.

Kaiser-Meyer-Olkin sampling adequacy measure (.85) and Bartlett's test of sphericity (χ^2^ = 4798.87, p < .001) supported the feasibility of exploratory factor analysis. In the Cattell's scree plot, only three factors were located to the left of the first elbow (Figure 2). The first is represented by the uncaring dimension (λ = 4.86, explained proportion of the variance: 14.22%), the second by the unemotional factor (λ = 2.27, 13.80%), and the third, by the callousness feature (λ = 1.73, 8.96%). The cumulative explained proportion was 36.98%.

Rotated factorial loadings are presented in Table 3. After applying the selection criteria, every item of the unemotional dimension was retained. In contrast, the callousness factor exhibited the highest number of items with inadequate statistical properties. The following questions were disregarded: Item 2 (maximum loading: .26 and minimum difference with other factors loadings: .11), item 5 (maximum loading .38 and minimum difference .16), item 7 (maximum loading .37 and minimum difference .15), item 8 (minimum difference .14 and misclassified as uncaring), item 10 (maximum loading .23, minimum difference .11 and misclassified as unemotional), item 12 (misclassified us unemotional), item 20 (minimum difference .01 and misclassified as uncaring).

**Table 3:** Rotated factorial loadings matrix for ICU items.

Items		Factor 1	Factor 2	Factor 3	H^2^	U^2^
*Uncaring*						
23.	Tenacity	0.765	0.013	-0.018	0.586	0.414
15.	Perseverance	0.739	0.072	0.059	0.555	0.445
3.	Concern for school duties	0.678	0.067	0.027	0.464	0.536
16.	Pride	0.539	0.294	0.171	0.406	0.594
17.	Affective intention	0.536	0.190	0.218	0.371	0.629
24.	Kindness	0.470	0.157	-0.032	0.247	0.753
13.	Fallibility	0.446	0.153	-0.034	0.223	0.777
8.	Affective interest∗	0.444	0.308	0.173	0.322	0.678
20.	Dedication∗	0.410	-0.048	0.405	0.335	0.665
5.	Guilt∗	0.379	0.223	0.142	0.214	0.787
*Unemotional*						
22.	Affective dissimulation	-0.008	0.757	0.178	0.604	0.396
19.	Emotionality	0.216	0.731	-0.108	0.593	0.407
12.	Coldness and disinterest∗	-0.044	0.687	0.236	0.529	0.471
1.	Affective expression	0.230	0.683	-0.055	0.523	0.477
14.	Affective identification	0.104	0.661	-0.103	0.459	0.541
6.	Emotional display	0.019	0.609	0.181	0.404	0.597
10.	Emotional self-control∗	-0.086	0.230	0.124	0.076	0.925
*Callousness*						
4.	Utilitarianism	0.083	0.148	0.645	0.445	0.555
21.	Affective irrelevance	0.183	0.153	0.625	0.447	0.553
18.	Remorse	0.018	0.117	0.578	0.348	0.652
11.	Academic performance	0.239	-0.043	0.436	0.248	0.752
9.	Potential for conflict	0.043	0.102	0.401	0.173	0.827
7.	Punctuality∗	0.218	0.055	0.369	0.187	0.813
2.	Discernment∗	-0.219	0.065	0.255	0.117	0.883

∗ (removed from the adapted version), H^2^.(communality), U^2^.(1-H^2^, unicity).

**Figure 3:**
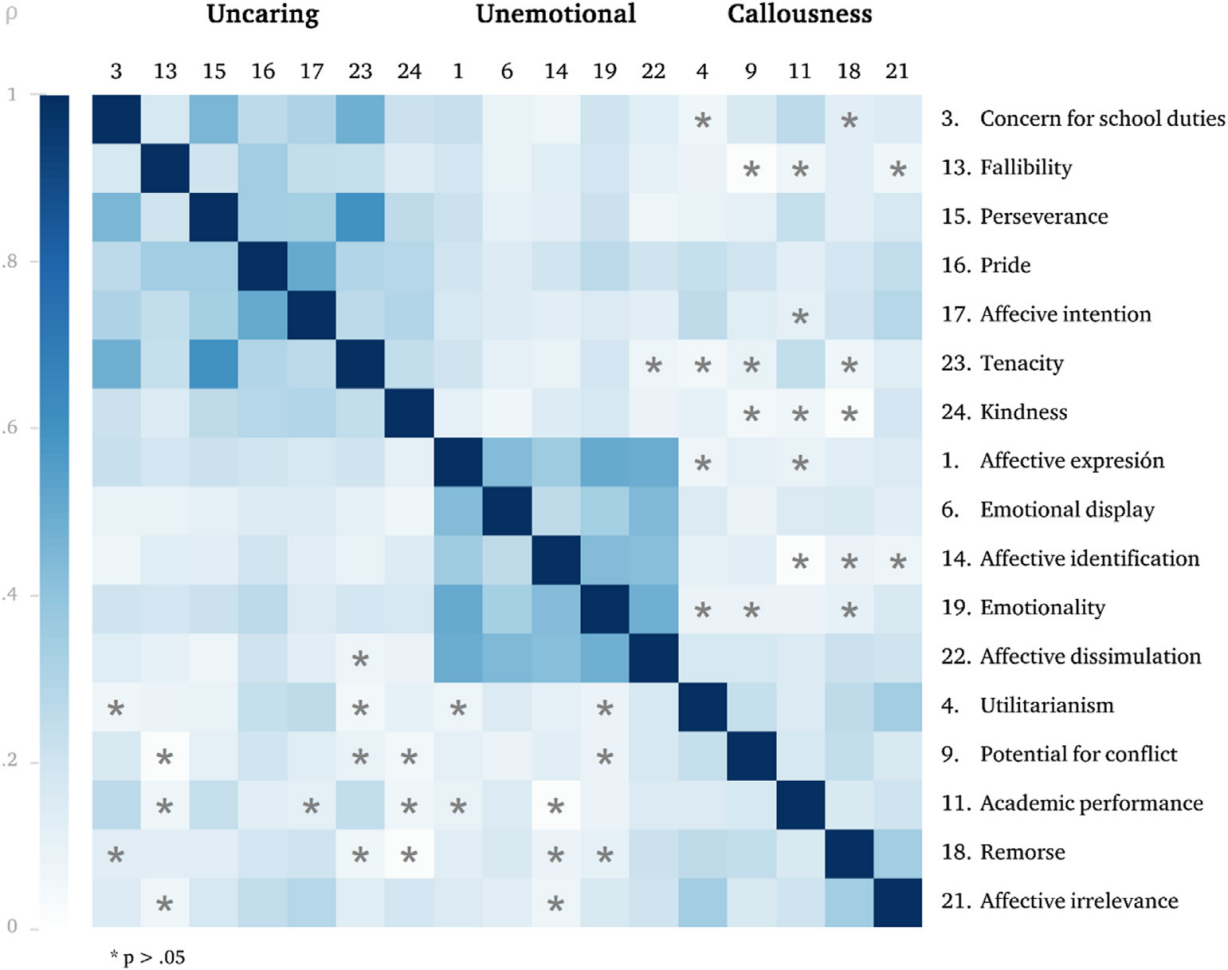
Matrix of intra-factor and inter-factor correlation coefficients between items.

Consequently, the factorial structure for the adapted version included 17 items with a total median score of 15 (IQR 11–19) and an acceptable reliability (α = .78, inter-item covariance .11) according to the defined methods. Factors were considered consistent (uncaring α = .75, unemotional α = .77, and callousness α = .55) and one-dimensional, and none of the items increased the internal consistency of its factor, after their isolated removal. Convergent and divergent validities were confirmed as intra-factor items correlation was higher than those inter-factor (Figure 3).

We built four different models to confirm the structural validity of the original 24-item version and the 17-item Colombian adaptation. Model A corresponds to the interpretation of CU as a one-dimensional construct, in order words, these traits are related to each statement, without the intermediation of the three factors. On the contrary, in model B, only the three dimensions are considered with an expected covariance, but a higher-level latent variable for CU is not included. In both models C and D, the construct of CU and its three factors is represented, but they differ as C corresponds to a bifactor approach (where there is no explicit relationship between CU and its factors, and therefore the items are associated with the four latent variables) while in D, a direct relationship between CU and the 17 statements is ruled out since the structure is hierarchical through the concepts of uncaring, unemotional, and callousness.

The adapted instrument showed a better fit than the original ICU in the Colombian population. None of the proposed models exhibited a statistical adjustment (p < .001) close to that of the theoretical saturated model (incorporating all the possible latent variables). In the goodness of fit analysis, model A had the highest AIC, BIC, RMSEA, and SRMR, exhibiting poor CFI and TLI indices. Furthermore, the B model structure is superior to the sole CU concept. When comparing C (bifactor) and the D (hierarchical) models, the former exhibited a lower value of χ^2^ (183.235, 101 degrees of freedom), and a reduction in AIC and BIC; the rest of the goodness of fit statistics were also satisfactory (Table 4).

**Table 4:** Goodness-of-fit statistics for the different models.

Statistics	Model A	Model B	Model C	Model D
*Original inventory*				
χ^2^	1567.635	1014.400	585.355	1512.955
AIC	47503.356	46694.239	46220.503	46698.239
BIC	47734.031	46939.331	46576.128	46952.942
RMSEA	0.076	0.058	0.042	0.075
SRMR	0.088	0.071	0.054	0.072
CFI	0.808	0.888	0.948	0.723
TLI	0.790	0.876	0.936	0.691
*Adapted version*				
χ^2^	911.270	317.044	183.235	622.497
AIC	33510.870	32603.660	32313.439	32607.660
BIC	33674.265	32781.472	32399.822	32795.083
RMSEA	0.086	0.044	0.030	0.070
SRMR	0.096	0.055	0.041	0.058
CFI	0.811	0.952	0.980	0.843
TLI	0.784	0.944	0.973	0.812

**Figure 4:**
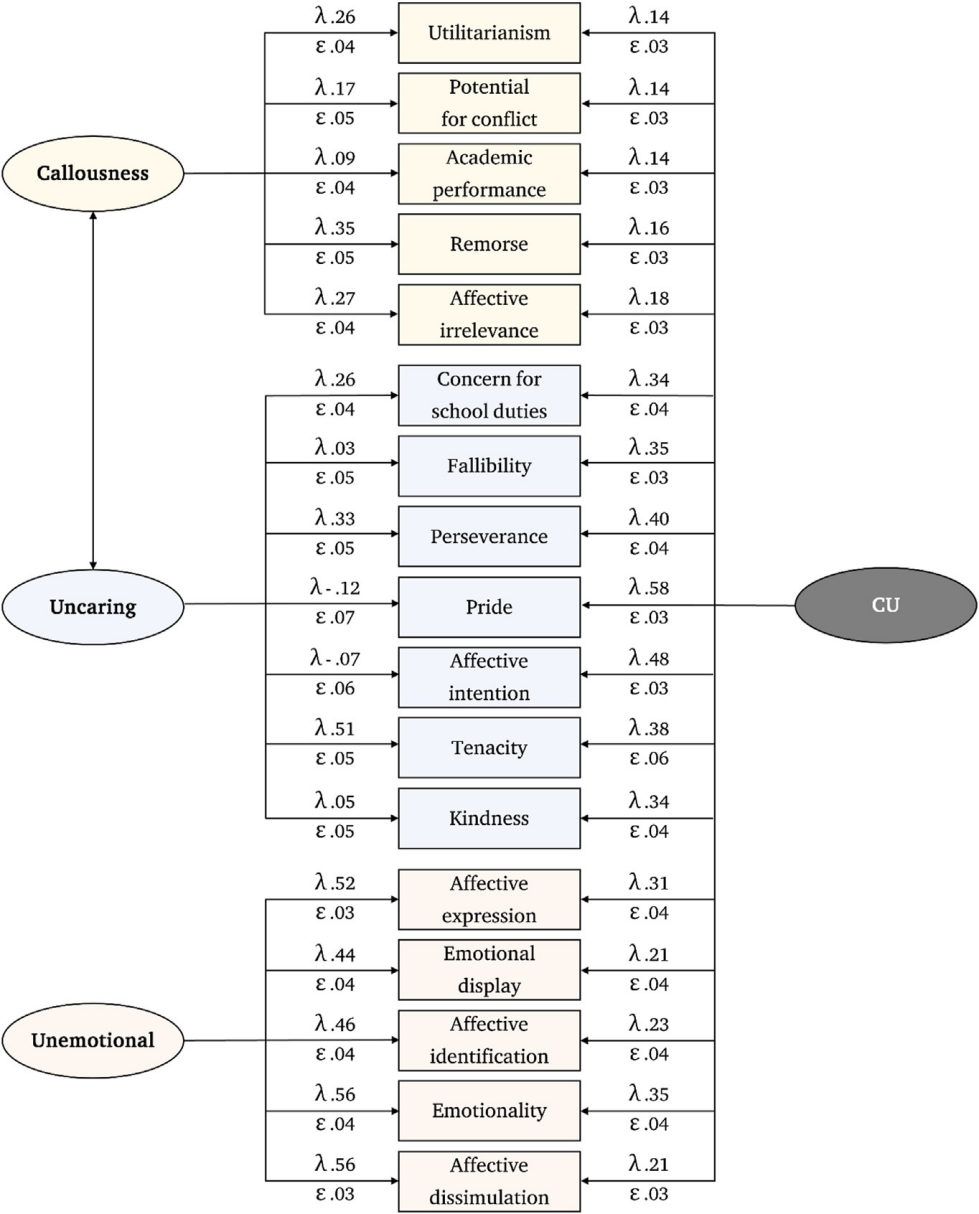
Structural model of the Colombian adapted version of the ICU by Frick.

Model C with 17-items was identified as the best approach (adjusted goodness of fit index .98) and showed comparable consistency and factorial structure when compared to the original instrument. As the covariances between unemotional and uncaring/callousness were not statistically significant, they were subsequently eliminated from the model (Figure 4). Items 13, 16, 17, and 24 did not contribute significantly to the uncaring factor but did so for the general concept of CU. Evaluation of the model in females and males demonstrated similar factorial structure and goodness of fit statistics. Metric, scalar, and strict invariance tests were consistent with equivalent factorial loadings, intercepts, and residual variances (Table 5).

**Table 5:** Study of invariance of the structural model across females and males.

Invariance	χ^2^	(p value)	dof	χ^2^/dof	RMSEA	CFI	TLI	SRMR
Configural	17.127	(0.079)	-	-	0.008	-0.005	-0.006	-0.006
Metric	28.322	(0.553)	30	0.944	-0.008	0.018	0.031	0.004
Scalar	19.987	(0.098)	13	1.462	0.000	-0.003	0.000	0.001
Strict	15.355	(0.569)	17	0.903	-0.001	0.001	0.004	0.002

### 3.5 Criterion validity

The original median ICU total score was 20 (IQR 15–26) for the neurotypical population, 25 (IQR 19–34) for the internalizing behavior arm, and 26 (IQR 19–34) for patients with externalizing disorders. For the 17-item adapted version, medians were 15 (IQR 11–19), 19 (IQR 14–26), and 19 (IQR 14–24), respectively. Statistically significant differences (χ^2^ = 33.48, 2 degrees of freedom) were identified between the neurotypical group and the individuals with internalizing (p < .001) and externalizing (p < .001) behaviors.

In comparison to controls, new subtotal scores by group indicated that participants with internalizing conduct are characterized by higher unemotional traits (median score: 10 IQR 8–12, χ^2^ = 23.81, 2 degrees of freedom, p < .001), those with externalizing disorders more commonly demonstrate uncaring behaviors (9 IQR 6–12, χ^2^ = 26.19, 2 degrees of freedom, p < .001), and both have higher callousness features (2 IQR 0–4, χ^2^ = 17.23, 2 degrees of freedom, p < .050).

By including the dimensions as regressors, the callousness factor was removed from the logistic multinomial model after the stepwise procedure. As evidenced in the posthoc comparisons, the unemotional dimension had a positive association only with the internalizing group (Logit coefficient 0.18, 95% CI 0.09–0.28, p < .001); the opposite occurred for uncaring, which was associated with the externalizing arm (Logit coefficient 0.16, 95% CI 0.08–0.23, p < .001). There were differences for the female sex, which supported the presence of internalizing behaviors (Table 6).

**Table 6:** Multinomial regression defining neurotypical participants as the reference population.

Regressor	Coefficient	95% CI		Error	p value
**Internalizing conduct**					
Female sex	1.275	0.660	1.889	0.314	<.001
Age (years)	0.606	0.270	0.942	0.171	<.001
School grade	-0.524	-0.906	-0.142	0.195	0.007
Unemotional	0.184	0.090	0.277	0.048	<.001
Uncaring	0.044	-0.039	0.127	0.042	0.296
Constant	-9.614	-12.172	-7.056	1.305	<.001
**Externalizing conduct**					
Female sex	-0.952	-1.632	-0.271	0.347	0.006
Age (years)	0.777	0.487	1.067	0.148	<.001
School grade	-0.820	-1.158	-0.482	0.172	<.001
Unemotional	-0.020	-0.113	0.072	0.047	0.663
Uncaring	0.156	0.079	0.233	0.039	<.001
Constant	-7.721	-9.738	-5.705	1.029	<.001

Finally, and due to the apparent usefulness of the original ICU total score (OR 1.07, 95% CI 1.04–1.10, p < .001), we determined 25 as the best cut-off value to discriminate neurotypical from externalizing conduct (sensitivity 54.69%, specificity 69.77%, and area under the ROC curve .67, 95% CI .60 - .74). For the adapted version, total score was associated with externalizing conduct (OR 1.09, 95% CI 1.05–1.13, p < .001) and the best cut-point was set at 22 (sensitivity 35.94%, specificity 85.38%, area under the ROC curve .65, 95% CI .58 - .72). Correct classification occurred in 82.11% and positive/negative likelihood ratios were 2.46/0.75.

Total validities were .84 for the original instrument and .83 for the adapted version.

## 4 Discussion

The present study aimed to adapt and validate the Inventory of Callous-Unemotional Traits (ICU) in a multi-centric Colombian sample of children and adolescents. The results pointed out a good content validity according to the participant judges, but an important confusion when categorizing items into callousness or uncaring. The consistency of both the total score and three subscales was similar to that reported in the literature. Items related to discernment (2), guilt (5), punctuality (7), affective interest (8), emotional self-control (10), coldness and disinterest (12), and dedication (20) were disregarded obtaining a 17-item version that fits the best in the shape of a sex-invariant bifactor model where each item is correlated with its dimension and the general CU construct. Criterion validity analysis detailed a good specificity of the instrument with significant differences in the total score between neurotypical participants and those with externalizing and internalizing conditions. The second group was characterized by higher uncaring characteristics and the third, by important levels of unemotionality.

In the process of validating the 24-item version, we maintained 17 items that exhibited good statistical results in the factor analysis according to the methods described. To date, 82% of studies validating the ICU have applied the 24-items version but there are exceptions, as the one from Mexico (Amador et al., 2017) and others with 23 (Kimonis et al., 2008, 2014; López-Romero et al., 2015b), 22 (Pechorro et al., 2019) or 12 (Hawes et al., 2014; Pechorro et al., 2016) items. A common finding between our sample and the published literature is the lack of fit of items 2 and 10 (López-Romero et al., 2015b; Pechorro et al., 2019). The most drastic modification resulted from the work by Hawes et al. (2014) on the parents’ version of the ICU. They examined the factor structure and deleted several items through item-test correlations (1, 2, 10, 14, 19, and 22) and a 2PL item-response theory model (3, 7, 13, 15, 20, and 23). At the end, only the callous and uncaring dimensions persisted as all but one item (6) of unemotionality were disregarded. Our adaptation of the instrument is in accordance with omitting items 2, 7, 10, and 20. It is important to highlight that the number of items established on each population does not seem to alter the internal consistency of the total score (α = .77 - .80) or the subscales (Deng et al., 2019).

The unemotional dimension in the Colombian sample was found to be more consistent than the callousness and uncaring ones. Likewise, we calculated a significant correlation coefficient among the three subscales, but a high number of the unemotional factor items were not associated with those of callousness. Evidence in the literature shows that the unemotional subscale is less reliable and weakly correlated with the general CU construct and the callousness and uncaring factors (Ciucci et al., 2014; Ezpeleta et al., 2013; Cardinale and Marsh, 2020). Others have considered it inconsistent because of its lack of relationship with psychopathic personality traits and conduct problems (Romero and Alonso, 2017) and even a multivariate genetic model suggested it does not share the same phenotype and genotype as the other two factors (Henry et al., 2016). Despite of these, Deng et al. (2019) found in their meta-analysis that the unemotional dimension seemed more reliable in non-English speaking populations (α = .71 95% CI .68 - .71 vs. α = .64 95% CI .59 - .69). The consistency reached a Cronbach's α of .87 in the Portuguese population investigated by Pechorro et al. (2014b). Similar estimates to ours have been informed in Spain (López-Romero et al., 2015b), Germany (Benesch et al., 2014), Belgium (Roose et al., 2013), Denmark (Kongerslev et al., 2015), Sweden (Thornberg and Jungert, 2017), and China (Luo, 2016), all with α > .75.

A group of researchers has suggested as well that factorial loadings for callousness and uncaring items differ not because each of them constitutes a single dimension, but due to a bias produced by wording (most of the callousness items are worded positively whereas uncaring items are predominantly negative) (Cardinale and Marsh, 2020). This could partially explain why the content judges were not able to correctly distinguish among items of the two dimensions. If the callousness and uncaring dimensions were eventually representing the same construct, and the unemotional items do not contribute to CU as a whole, it could be reasonable that most of the studies, such as ours, have found a bifactor model and not a hierarchical one.

External validation of the ICU total and subtotal scores in the function of externalizing outcomes has resulted in medium effect size associations except for the unemotional subscale. On the contrary, associations of this factor with internalizing disturbances were positive but small (Cardinale and Marsh, 2020). Although marginally significant, multinomial logistic modeling applied to the Colombian sample demonstrated that unemotionality is positively associated with internalizing disorders but is opposed to the presence of externalizing behavior. Complementarily, the coefficient of association with the uncaring dimension is higher for participants with externalizing disorders. Both groups scored higher than neurotypical participants in callousness.

Our study has multiple strengths, such as including a large sample from different cities of the country, in an attempt to account for the cultural diversity within the national population. There was a rigorous linguistic adaptation of the ICU, and the final retro-translation was quantitatively compared with the original version showing a high level of similarity. Validity of the instrument was assessed globally and determined not only the quality of the ICU construct but also of its content and criterion. To the best of our knowledge, no recent validation has exposed the difficulties to conceptually classifying callousness and uncaring items by content judges. It is important to note that our study also has some limitations: first, we were unable to implement a nation-wide randomized schools sampling, as permission from other institutions could not be obtained in time due to the current public health settings that forbidden and then limited the presence of students at schools. In that sense, it was not possible to verify the informed diagnoses of externalizing and internalizing conditions using, for example, a semi-structured psychiatric interview. Second, exploring concurrent validity with other instruments that address psychopathic traits was not possible, because the availability of time for applying (and re-applying) the test was restricted, and there are not validated instruments to appraise CU in Colombian children and adolescents with no criminal background.

This study provides a validated adaption to the Colombian Spanish of the most commonly used instrument to assess callous-unemotional traits in children and adolescents. The results preliminary support the content, construct, and criterion validity of the ICU for characterizing callous-unemotional traits. Additionally, the structure of the instrument is invariable by sex.

Research on CU should be expanded, and further studies need to be carried out to validate the inventory in underage offenders and clinical samples of children suffering from neurodevelopmental and disruptive behavior disorders (i.e., autism, attention-deficit/hyperactivity disorder, conduct disorder, etc.). Although most validations are based on ICU self-report, future studies ought to estimate if the agreement with reports by teachers and parents is satisfactory. We expect that the adaptation of the ICU in Colombia could be of use for upcoming research focused on the relationship between these traits and socio-cultural and neurobiological factors related to psychopathy in order to facilitate early diagnosis and timely interventions.

## Declarations

### Author contribution statement

Olber Eduardo Arango-Tobon: Conceived and designed the experiments; Performed the experiments; Wrote the paper.

Gabriel David Pinilla-Monsalve: Analyzed and interpreted the data; Contributed reagents, materials, analysis tools or data; Wrote the paper.

Anyerson Stiths Gómez-Tabares; Andrés Mauricio Grisales Aguirre: Analyzed and interpreted the data; Wrote the paper.

César Andrés Carmona-Cardona: Performed the experiments; Wrote the paper.

### Funding statement

This work was supported by Luis Amigó Catholic University (05020299119).

### Data availability statement

Data will be made available on request.

### Declaration of interest’s statement

The authors declare no conflict of interest.

### Additional information

No additional information is available for this paper.
